# α1-Antitrypsin attenuates acute rejection of orthotopic murine lung allografts

**DOI:** 10.1186/s12931-021-01890-x

**Published:** 2021-11-17

**Authors:** Tomoyuki Nakagiri, Sabine Wrenger, Kokilavani Sivaraman, Fabio Ius, Tobias Goecke, Patrick Zardo, Veronika Grau, Tobias Welte, Axel Haverich, Ann-Kathrin Knöfel, Sabina Janciauskiene

**Affiliations:** 1 Department of Cardiothoracic, Transplantation, and Vascular Surgery, Hannover Medical School, Hannover, Germany; 2 Department of Respiratory Medicine, Hannover Medical School, Hannover, Germany; 3 Biomedical Research in Endstage and Obstructive Lung Disease Hannover (BREATH), Member of the German Center for Lung Research (DZL), Hannover, Germany; 4 Department of General and Thoracic Surgery, Laboratory of Experimental Surgery, Justus-Liebig-University Giessen, German Center for Lung Research, Giessen, Germany

**Keywords:** Lung transplantation, Acute allograft rejection, Mouse orthotopic single lung transplantation model, Primary graft dysfunction, Alpha1-antitrypsin

## Abstract

**Background:**

α1-Antitrypsin (AAT) is an acute phase glycoprotein, a multifunctional protein with proteinase inhibitory, anti-inflammatory and cytoprotective properties. Both preclinical and clinical experiences show that the therapy with plasma purified AAT is beneficial for a broad spectrum of inflammatory conditions. The potential effects of AAT therapy have recently been highlighted in lung transplantation (LuTx) as well.

**Methods:**

We used a murine fully mismatched orthotopic single LuTx model (BALB/CJ as donors and C57BL/6 as recipients). Human AAT preparations (5 mg, n = 10) or vehicle (n = 5) were injected to the recipients subcutaneously prior to and intraperitoneally immediately after the LuTx. No immune suppressive drugs were administered. Three days after the transplantation, the mice were sacrificed, and biological samples were assessed.

**Results:**

Histological analysis revealed significantly more severe acute rejection in the transplanted lungs of controls than in AAT treated mice (p < 0.05). The proportion of neutrophil granulocytes, B cells and the total T helper cell populations did not differ between two groups. There was no significant difference in serum CXCL1 (KC) levels. However, when compared to controls, human AAT was detectable in the serum of mice treated with AAT and these mice had a higher serum anti-elastase activity, and significantly lower proportion of Th1 and Th17 among all Th cells. Cleaved caspase-3-positive cells were scarce but significantly less abundant in allografts from recipients treated with AAT as compared to those treated with vehicle.

**Conclusion:**

Therapy with AAT suppresses the acute rejection after LuTx in a mouse model. The beneficial effects seem to involve anti-protease and immunomodulatory activities of AAT.

## Background

Lung transplantation (LuTx) is an established therapy for end-stage lung diseases. In recent years the number of lung transplants has increased worldwide, and patient survival worldwide increased from a median of 4.3 years (1990–1998) to 6.5 years (2009–2016) [1]. However, as compared to other solid organ transplantations, the outcome after LuTx does not reach a satisfying result [2]. According to the international society of heart and lung transplantation (ISHLT), the 5 years survival after LuTx is about 59% whereas that after heart transplantation is 77%. The main reason for this poor outcome is a chronic lung allograft dysfunction (CLAD) [3]. The bronchiolitis obliterans syndrome (BOS) is the most common cause of death, its incidence is over 70% of the CLAD [4]. There are events strongly contributing to the development of BOS, including primary graft dysfunction (PGD), cytomegalovirus infection and acute lung rejection [5]. Different studies reported that patients with BOS have diminished levels of Tregs, a subset of CD4+ T cells, and that the misbalance between Tregs and Th17 (a CD4+ T-cell subset producing interleukin-17) may predict a risk of developing BOS [6]. In agreement, we reported that the higher frequencies of regulatory T cells (T regs) correlate with lower risk of developing BOS [7, 8].

The prevention of acute lung rejection is one of the strategies against CLAD development. Because of a strong immune system of the lungs, the recipients after LuTx receive high doses of immune suppressions, especially calcineurin inhibitors [5], which may cause various complications, including damage of renal function, infections, and development of tumors [9, 10]. Therefore, the development of new approaches weaning LuTx patients from immunosuppression are warranted.

Alpha 1-antitrypsin (AAT) is an acute phase glycoprotein, a major human serine protease inhibitor [11] and a broad regulator of immune and inflammatory responses. It is well known that severe inherited AAT deficiency predisposes to an early-onset pulmonary emphysema, which is a common indication for LuTx [12, 13]. Several preclinical and clinical studies have shown that therapy with plasma purified AAT is not only beneficial for emphysema patients with inherited AAT deficiency, but also useful for other inflammatory and immune-mediated conditions [14]. Results from various cell culture and animal studies show that the therapy with human AAT limits cell activation and tissue injury [11]. It is clear, AAT is not only a major inhibitor of serine proteases but also acts as an inhibitor of caspases and apoptosis, as a modulator of pro- vs anti-inflammatory cytokine balance, and as an enhancer of immunologic tolerance [15]. Studies based on animal models reported that AAT can promote tolerance by downregulating early inflammation and contributing to the induction of regulatory T cells (Tregs) [16–18]. These studies have led to a new understanding that AAT is a modulator but not a suppressor of local and systemic inflammatory responses. Therefore, there is a strong interest in determining whether the diverse immune-modulatory effects of AAT therapy and its well-established tolerability in patients with inherited AAT deficiency might be useful in lung transplantation. In fact, scientists reported that human plasma AAT improves lung function in rodent models of lung ischemia–reperfusion and lung transplantation [19]. For example, in a rat lung transplantation model, authors demonstrated that priming the donor lung with AAT and the post-transplantation treatment of the recipient with AAT, reduces acute lung injury and necrosis [20]. In a pig model, the treatment of recipients with AAT before lung transplantation significantly improved graft survival [21]. A phase 2 clinical trial that uses intravenous AAT therapy to prevent primary graft dysfunction in lung transplant is underway at the University Toronto, Canada [15]. It is also important to point out that lung grafts perfused and stored in a colloid-based electrolyte solution (Perfadex) supplemented with AAT exhibited significantly less inflammation-mediated damage after transplantation as compared to the lungs stored in Perfadex alone [22]. Nowadays, ex vivo organ perfusion (EVOP) techniques have been developed to increase the graft acceptance rates and to improve the clinical outcomes [23]. Remarkably, for 24 h preserved pig donor lungs with EVOP containing AAT showed better physiologic function and lower cell death than lungs in EVOP without AAT [24]. Taken together, above findings encourage future research on AAT therapy as a useful in LuTx.

Recent studies reported that the administration of exogenous AAT prevents ischemia–reperfusion-induced lung injury (IRI) in vivo [19, 22], the major cause of PGD and, as is mentioned above, one of the risk factors for CLAD development. Herein we used a mouse orthotopic lung transplantation (OLTx) model to investigate the putative usefulness of AAT to prevent acute lung rejection after LuTx.

## Methods

### Animals

The study protocol was approved by the Lower Saxony State Office for Consumer Protection and Food Safety (approval No. 16-2166). Animal experiments were performed according to the German Animal Welfare Act as well as the European Guidelines for Animal Experiments (Directive 2010/63/EU). BALB/CJ and C57BL/6 mice were bred at the Central Animal Facility of Hannover Medical School under specified pathogen-free conditions, 12 h day–night rhythm, 20 °C, access to water and standard chow *ad libidum*. Male mice weighing 25–30 g (12–16 weeks old mice, AAT treated mice: n = 10, control mice: n = 5) were used for allogenic transplantation.

### Mouse orthotopic single lung transplantation (OLTx)

Our OLTx technique is a modification of previously published techniques [25, 26] and was recently detailed elsewhere [27]. Briefly, donor and recipient mice were subcutaneously treated with butorphanol (Zoetis Deutschland GmbH, Berlin, Germany; 1 mg/kg body weight), intubated (20G indwelling catheter), connected to a pressure-controlled ventilator (UNO micro-ventilator, UNO Röstvaststaal, Zevenaar, Netherlands) and ventilated with O_2_ containing 1.5% isoflurane (Abbvie Inc. North Chicago, Illinois) at a rate of 120 breaths per min, a pressure of 12 cm H_2_O, and a positive end expiratory pressure of 2 cm H_2_O. After laparotomy and thoracotomy, donor lungs were flushed via the right ventricle with 5 ml Perfadex® (XVIVO, Göteborg, Sweden) containing heparin (ratiopharm GmbH, Ulm, Germany; 0.1 U/ml). The heart–lung block was excised and stored on ice. Left thoracotomy of the third intercostal space was performed in recipients and the pulmonary artery and vein were separated from the left main bronchus and ligated. First, the vein was anastomosed using a 22G cuff, then the bronchus was anastomosed using 18G cuff and finally, the artery was anastomosed with a 27G cuff. Central ligations were released, and the native left lung was removed. After visually controlling the perfusion and ventilation of the allograft, the chest was closed, isoflurane was stopped, and the animals were weaned from the ventilator. Allograft recipients obtained two subcutaneous injections of butorphanol (1 mg/kg body weight) and one injection of ciprofloxacin (Fresenius Kabi Deutschland GmbH, Bad Homburg, Germany; 7 mg/kg body weight) per day until the end of the experiment. No immunosuppressive medicaments were given. In about 40% of the experiments, animals died during surgery; these experiments were excluded from the study.

### Administration of AAT

**Fig. 1:**
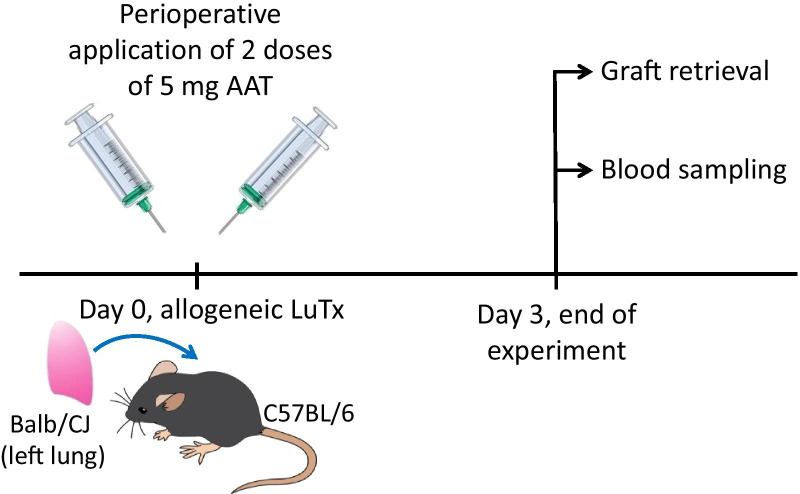
Study protocol. Donor lungs were harvested from BALB/CJ mice. Alpha1-antitrypsin (AAT) was administrated subcutaneously to the recipient before the transplantation. A harvested lung was transplanted into a C57BL/6 mouse orthotopically. After the transplantation, the second dose of AAT was immediately injected intraperitoneally. Control mice received no AAT, but the same volume of saline vehicle. The transplanted mice received analgesic and antibiotic, but no immune suppression. Three days after the transplantation, the mice were sacrificed, and whole blood and lung lobes were harvested

The design of the study is summarized in Fig. 1. Plasma purified human AAT (Respreeza, CSL Behring, Marburg, Germany or Prolastin, Grifols, Barcelona, Spain) was used in this study after a buffer exchange to sterile Dulbecco’s phosphate buffered salt solution (PBS, Gibco Thermofisher Scientific, Waltham, Massachusetts, USA), by using 10 K molecular weight cut-off centrifugal filter columns (Thermofisher Scientific). We purposely used two commercial preparations of AAT approved for administration to patients in Germany. After exchanging buffer, we expected to see similar short-term effects of both AAT preparations in vivo. If AAT expresses specific anti-protease, anti-inflammatory or other putative effects in vivo, this should be visible regardless of the preparation. Two doses of 5 mg of AAT, each, were administered to recipient mice, one subcutaneously 20 min before LuTx, shortly after induction of anesthesia and the second intraperitoneally immediately after LuTx. Control animals obtained the same volume of 0.9% sterile saline (n = 5).

### Blood sampling and graft retrieval

On postoperative day 3, allograft recipients were anesthetized and ventilated as described above. After evaluation of the macroscopic appearance of the allograft, recipients were sacrificed by exsanguination via the inferior caval vein. For flow cytometry, one drop of blood was mixed with 1 ml PBS (Bio and Sell GmbH, Nürnberg, Germany) containing 2 U/ml heparin and about 0.7 ml blood were used for serum preparation. Thereafter, the circulation was flushed with 5 ml saline and the appearance of the transplanted lungs was evaluated macroscopically. Subsequently, the lungs and the heart were removed as a complete unit.

### Histopathology

The harvested tissue block was fixed by immersion in 3.7% buffered formalin overnight. Thereafter, lungs were cut transversely and embedded in paraffin. Paraffin sections were dewaxed, rehydrated, and stained with hematoxylin–eosin (HE).

According to the consensus classification for acute rejection of human lung transplants of the ISHLT [28], lung sections were evaluated by a scientist blinded to the experimental groups.

### Immunohistochemistry

AAT immunoreactivity and activated caspase-3 in the transplanted lungs were evaluated using immunohistochemistry**.** Lung transplanted tissue sections were deparaffinized in xylene (Roth, Karlsruhe, Germany) for 15 min, afterwards incubated in xylene plus isopropanol solution (1:1 ratio) and then in 100% isopropanol immersion for 10 min respectively. Subsequently, tissue sections were hydrated by an ethanol gradient (100%, 90% and 70%). Antigen retrieval was achieved by heat induced epitope retrieval (HIER) method using 10 mM sodium citrate buffer (pH 6.0) at 85 °C for 45 min. Thereafter, endogenous peroxidases were blocked with 3% hydrogen peroxide (H_2_O_2_, Sigma-Aldrich, St. Louis, Missouri, USA) for 10 min at room temperature, followed by washing with PBS for three times (5 min each). Unspecific antigens were blocked with PBS containing 1% bovine serum albumin (Merck-Millipore, Burlington, Massachusetts, USA), 10% fetal calf serum (Gibco, Thermofisher Scientific, Waltham, Massachusetts, USA), and 0.3% Tween-20 (Sigma-Aldrich) for 1 h at room temperature. Post blocking, sections were incubated with rabbit polyclonal anti-cleaved caspase-3 primary antibody (Asp175, Cell Signaling Technology, Danwers, Massachusetts, USA) at 1:400 dilution or with rabbit polyclonal anti-human AAT (DAKO, Copenhagen, Denmark) at 1:50,000 for overnight at 4 °C in a humidifying chamber. Thereafter, sections were incubated with horseradish peroxidase (HRP) polymer (Gene Tex, Wien, Austria), for 45 min and with 3,3′-diaminodbenzidine (DAB, Zytomed, Bargteheide, Germany) for 15 min at room temperature. Sections were then counterstained with hematoxylin and mounted with eukitt quick hardening mounting medium (Sigma Aldrich) and analyzed under a light microscope. Images were taken with 40× or 100× objective using Leica DM750 microscope equipped with Leica ICC50 HD camera (Leica Microsystems, Wetzlar, Germany). Quantification of cleaved caspase-3 staining was done by counting the cleaved caspase-3 positive cells from 15 fields of each lung section.

### Flow cytometry

**Fig. 2:**
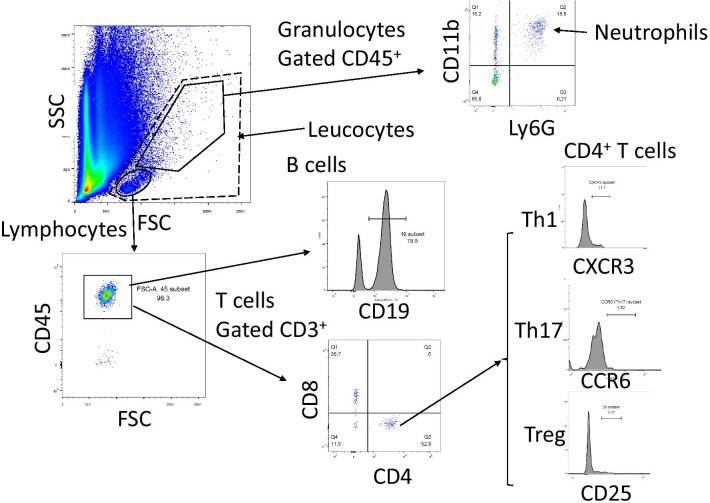
Gating strategy for immune cell analysis. Whole blood was diluted with PBS and stained with antibodies. Granulocytes were sorted from the granulocyte area and selected for neutrophils by using antibodies against CD45, CD11b and Ly6G. The percentage of neutrophils was calculated from the whole leukocytes. Lymphocytes were sorted from the lymphocyte area by expression of CD45. B cells were identified using against CD19 antibodies. The percentage of B cells from CD45-positive cells in the lymphocyte gate was calculated. Various T cell populations were selected using antibodies against CD3 and specific markers. The percentage of the cytotoxic T cells (as identified by CD8 expression) and the total T helper cell population (as identified by CD4 expression) from total CD3-expressing T cells was calculated. The percentages of Th1, Th17 and Tregs from CD4-expressing helper T cells were determined using the surface markers as specified in the figure

One drop of heparinized blood was mixed in one ml PBS containing heparin (2 units/ml). The general gating strategy is visualized in Fig. 2. All antibodies were obtained from BioLegend (San Diego, CA, USA). The blood was stained with fluorescently labelled antibodies to CD45 (APC, clone 30-F11), Ly-6G (FITC, clone 1A8), CD11b (PE/Cy7, clone M1/70) to identify neutrophils. Antibodies to CD45 and CD19 were used to stain B cells. T helper cells were identified with antibodies to CD45, to CD3 (PE/Cy7, clone 17A2), to CD4 (FITC, clone RM4-5), and to CD8 (PerCP/Cy5.5, clone RPA-T8). Subgroups were differentiated with antibodies to CD25 (Tregs; APC, clone PC61), to CXCR3 (Th1 cells; PerCP/Cy5.5, clone CXCR3-173) and to CCR6 (Th17 cells; PE, clone 29-2L17). The FACSAria flow cytometer (Becton Dickinson, Franklin Lakes, NJ, USA) was used.

### Serum anti-elastase activity

Anti-elastase activity of mice serum was measured photometrically as described previously [29]. Briefly, serum was pre-incubated with elastase at 37 °C in 0.1 M Tris buffer, pH 8. After 5 min, elastase substrate succinyl-Ala-Ala-Ala-*p*-nitroanilide (Sigma Aldrich, St. Louis, Missouri, USA) was added and the absorbance was followed for 3 min at 405 nm on Infinite M200 microplate reader (Tecan, Männedorf, Switzerland). Sample containing substrate and buffer alone was used for blank reduction. Elastase inhibition was calculated relative to samples containing only elastase and substrate.

### KC/CXCL1 serum concentration

KC/CXCL1 serum concentration was quantified by ELISA using mouse CXCL1/KC DuoSet (R&D Systems, Minneapolis, Minnesota, USA) according to the instructions of the manufacturer. Assay Range: 16–1000 pg/mL.

### Detection of serum AAT by Western blot

Equal amounts of serum were separated by gel electrophoresis using 10% SDS–polyacrylamide gels prior to transfer onto polyvinylidene difluoride (PVDF) membranes (Merck-Millipore, Burlington, MA, USA). Membranes were blocked for 1 h with 5% low fat milk (Carl Roth, Karlsruhe, Germany) followed by overnight incubation at 4 °C with polyclonal rabbit anti-human AAT (1:800) (DAKO A/S, Glostrup, Denmark). The immune complexes were visualized with anti-rabbit HPR-conjugated secondary antibodies (DAKO A/S) and enhanced by Clarity Western ECL Substrate (BioRad, Hercules, CA, USA). Images were acquired by using Chemidoc Touch imaging system (BioRad, Hercules, CA, USA) and processed using Image Lab version 5.2.1. software (Bio-Rad). The apparent molecular weight was determined using PageRuler Plus Prestained Protein Ladder (Thermofisher Scientific, Waltham, Massachusetts, USA) marker.

### Detection of human AAT by ELISA

Exogenously applied human AAT was measured in murine serum using a human Serpin A1 DuoSet ELISA lacking cross-reactivity for murine Serpin A1 (DY1268, R&D Systems, Bio-Techne, Wiesbaden, Germany), according to the manufacturer’s instructions. Serum samples were diluted 1:10,000. The optical density was determined using a microplate reader Infinite M200 (Tecan, Männedorf, Switzerland), measuring absorbance at 450 nm with the reference wavelength at 540 nm. All measurements were performed in duplicates.

### Statistical analyses

Data were statistically analyzed with GraphPad Prism 8, Version 8.4.3 (GraphPad Software, LLC). Groups of data were analyzed using two-tailed Mann–Whitney rank sum test. A p value of ≤ 0.05 was considered as statistically significant.

## Results

### Graft morphology

**Fig. 3:**
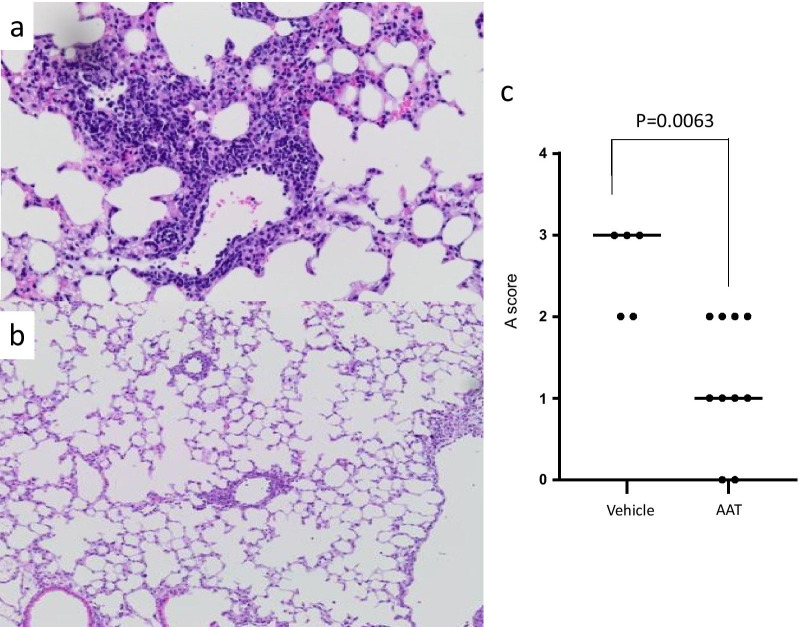
Histological assessment of allografts. Sections were stained with HE. **a** Left transplanted lungs of AAT treated mice had a normal appearance with shape edge (no edema), healthy normal color and good compliance. Microscopically, the lung had only slight inflammation around vessels (rejection score A1-2) without distraction of lung parenchyma. **b** The controls had a reddish and edematous graft lung with bad compliance. The microscopic view showed strong inflammation, not only around vessels, but also in the parenchyma with distraction. **c** According to the classification of the international society of heart and lung transplantation, the differences of the acute rejection degree showed a significance between control group and AAT treated mice (control vs. AAT group: median 3 vs. 1, p = 0.0063, respectively). The p-value was calculated using two-tailed Mann–Whitney rank sum test. A p value of ≤ 0.05 was considered as statistically significant

Early hallmarks of acute rejection of fully allogenic experimental grafts are seen within the first days after transplantation. Therefore, the morphology of ventilated allografts was evaluated macroscopically immediately after thoracotomy on the third postoperative day. In control mice treated with vehicle, the allografts had a reddish color, an edematous appearance and were not properly ventilated (n = 5, Fig. 3a). In contrast, when recipients were treated with AAT, the allografts had normal appearance and were nicely ventilated (n = 10, Fig. 3b).

Allograft histopathology of recipients treated with vehicle depicted hallmarks of moderate to severe acute rejection. Airways and blood vessels were surrounded by several layers of infiltrating leukocytes and alveolar septa were thickened (Fig. 3a). In contrast, the infiltrates were milder, and the histopathology of lung allografts from both groups of recipients treated with AAT was compatible with mild acute rejection (Fig. 3b). Scoring of allograft sections according to the ISHLT classification of acute rejection, which is the official guideline for the histopathological evaluation of human pulmonary grafts [28], revealed significant (p = 0.006) differences among allografts from recipients treated with vehicle (n = 5, median 3) and AAT (n = 10, median 1; Fig. 3c).

### Detection of AAT in recipient serum and allografts

**Fig. 4:**
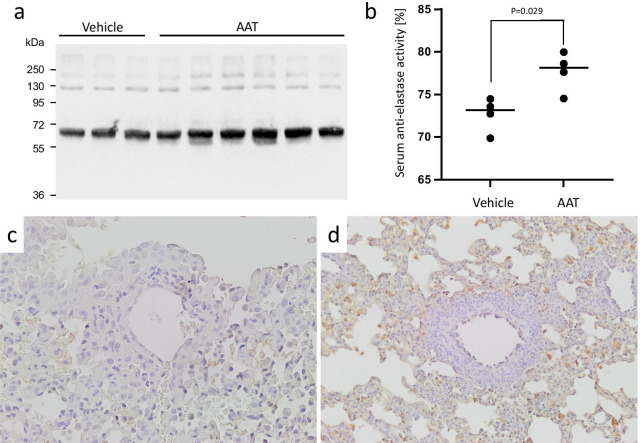
Detection of AAT in serum and lung tissue at day 3 after lung transplantation. **a** Using Western Blot, serum AAT levels were evaluated. AAT is a glycoprotein with a molecular mass of about 52 kDa. Sera from the AAT treated group showed remarkably thicker AAT bands, compared to those of the controls. **b** Serum anti-elastase activity reflects AAT functionality. Serum anti-elastase activity was evaluated spectrophotometrically. AAT-treated mice (n = 4) showed a significantly higher anti-elastase activity than controls: (n = 4): 72.7 ± 1.7 vs. 77.7 ± 2.0, p = 0.029. The p-value was calculated using two-tailed Mann–Whitney rank sum test. A p value of ≤ 0.05 was considered as statistically significant. **c**, **d** The immunohistochemical AAT staining showed remarkable difference between vehicle treated control mice (**c**) and AAT treated mice (**d**). Sections of graft tissue were stained with anti-AAT polyclonal antibody and HRP-DAB. Brown color indicates AAT. Cell nuclei were visualized by hematoxylin. Images were taken using 100× objective for (**c**) and 40× objective for (**d**) on a Leica DM750 microscope equipped with Leica ICC50 HD camera

Serum AAT of allograft recipients was detected on postoperative day 3 by Western blotting. The anti-AAT antibodies used in this study detect both, endogenous murine AAT, and exogenous human AAT (Fig. 4a). Serum AAT had an apparent molecular mass of about 65 kDa in all allograft recipients. In graft recipients treated with AAT (n = 6), the immunoreactivity was stronger compared to recipients treated with vehicle (n = 3). As expected, using a specific ELISA for the quantification of human AAT, we found human AAT in the serum of mice treated with AAT preparations [mean (SD), n = 6: 29.35 (14.9) µg/ml] but not in non-treated control mice. In accordance with increased AAT levels, a higher anti-elastase activity was observed in serum of graft recipients treated with AAT as compared to those treated with vehicle (Fig. 4b: median 79.1% vs. 73.1%, p = 0.029, respectively). This latter finding confirms the inhibitory activity of the administered AAT protein. Moreover, the presence of AAT in lung allografts was confirmed by immunohistochemistry (Fig. 4c, d).

### Activated caspase-3 in allografts

**Fig. 5:**
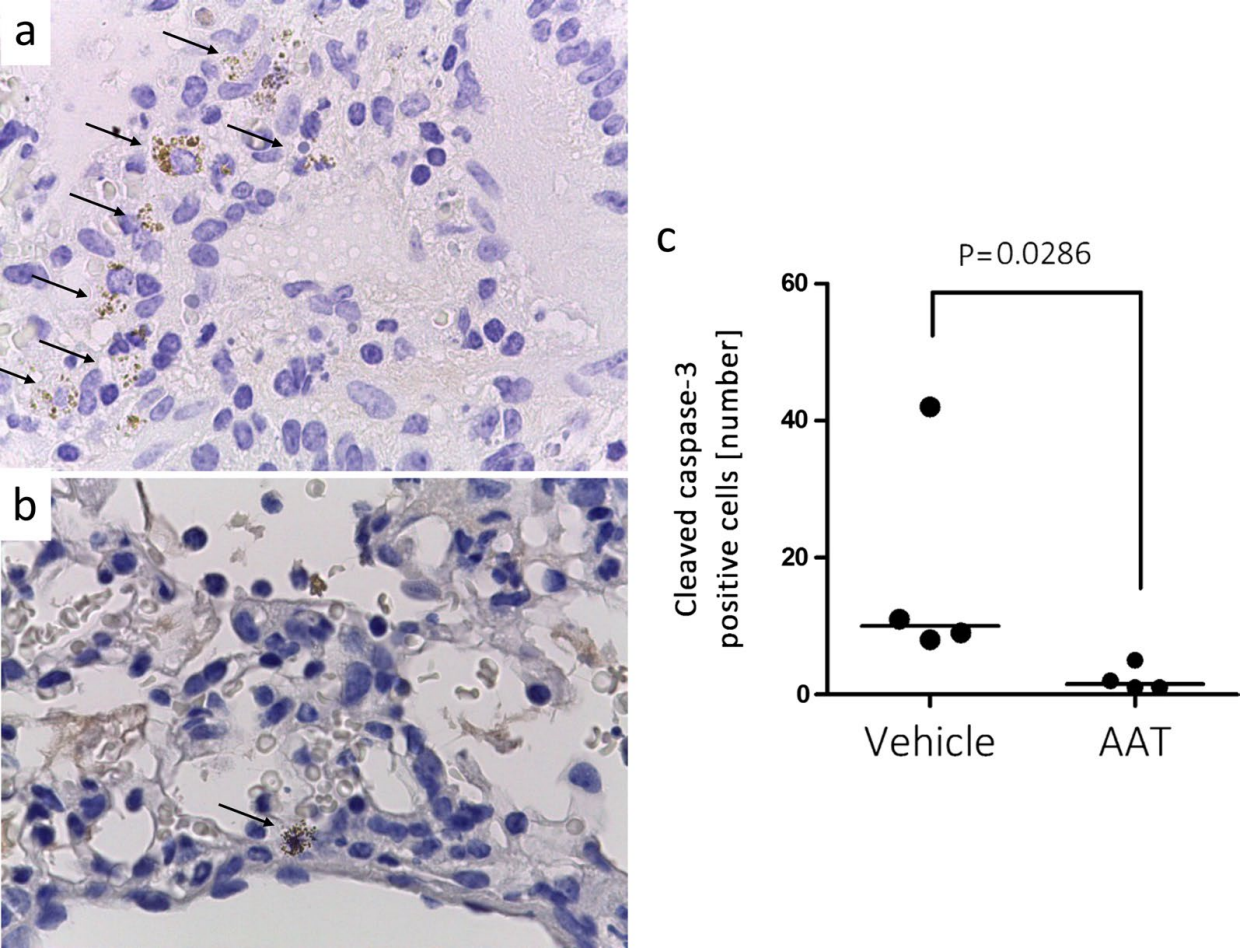
Detection of apoptosis via immunohistochemical analysis of cleaved caspase 3: Sections of graft tissue were stained with anti-cleaved caspase-3 polyclonal antibody and HRP-DAB. Nuclei were visualized by hematoxylin. **a** Graft tissue from a vehicle treated mice and **b** graft tissue form AAT-treated mouse. Arrows indicate cleaved-caspase 3 (brown color). Images were taken using 100× objective on a Leica DM750 microscope equipped with Leica ICC50 HD camera. **c** Positive cells were counted from 15 different fields of each specimen from four different recipients of each group. The p-value was calculated using two-tailed Mann–Whitney rank sum test. A p value of ≤ 0.05 was considered as statistically significant

To evaluate possible effects of AAT on apoptosis, cleaved-caspase-3 was stained immunohistochemically on allograft sections. Positive cells were scarce but significantly more cleaved-caspase-3-positive cells were detected in allografts from recipients treated with vehicle than in those treated with AAT (number of positive cells in 15 fields: vehicle (n = 4, median 10.0) vs. AAT (n = 4, median 1.5), p = 0.0268; Fig. 5).

### Analysis of allograft immune responses

**Fig. 6:**
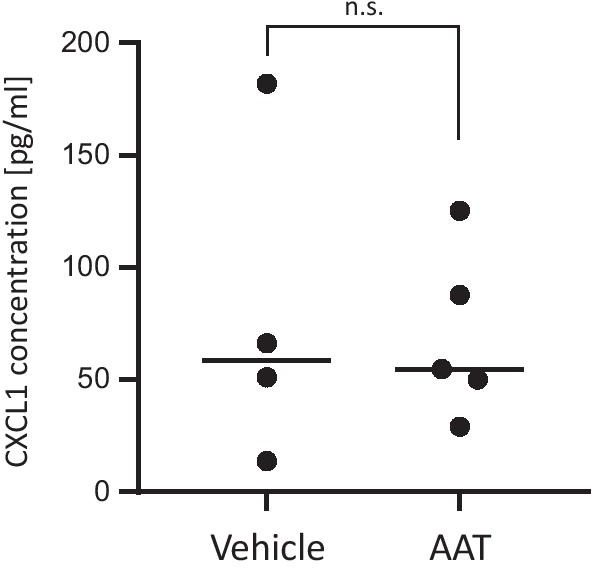
Analysis of KC/CXCL1 serum levels as a measure for the neutrophil contribution in allograft rejection. Serum CXCL1 concentrations as measured by ELISA did not differ among the experimental groups (vehicle, n = 4, median 58.7 vs. AAT, n = 5, median 54.7, p > 0.99). The p-value was calculated using two-tailed Mann–Whitney rank sum test. A p value of ≤ 0.05 was considered as statistically significant

**Fig. 7:**
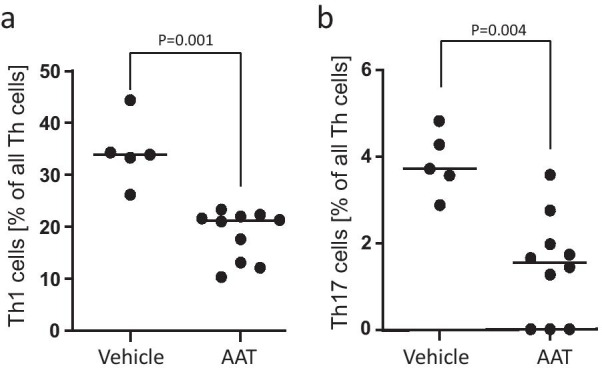
Flow cytometry analysis of peripheral blood leukocytes to detect the Th1 and Th17 populations of lymphocytes. For gating strategy see Fig. 2. **a** Significant differences were detected in the Th1 cells between vehicle treated control mice (n = 5) and AAT treated mice (n = 10): vehicle vs. AAT group (percentage of all Th cells): p = 0.001. **b** Significant differences were detected in the Th17 cells between vehicle treated control mice (n = 5) and AAT treated mice (n = 10); vehicle vs. AAT group (percentage of all Th cells, p = 0.004). The p-values were calculated using two-tailed Mann–Whitney rank sum test. A p value of ≤ 0.05 was considered as statistically significant

As measured by ELISA, the concentrations of serum CXCL1 (KC), a major chemoattractant and activator of neutrophils, did not differ among the experimental groups: (vehicle, n = 4, median 58.7 pg/ml vs. AAT, n = 5, median 54.7 pg/ml, p > 0.99, Fig. 6). To identify immune cell populations, the proportion of blood lymphocyte subsets and neutrophils was analyzed in allograft recipients by flow cytometry on postoperative day 3. As shown in Table 1, the proportion of neutrophil granulocytes (as identified by CD11b and Ly6G expression) from CD45-positive leukocytes, of B cells (as identified by CD19 expression) from CD45-positive cells in the lymphocyte gate, of the cytotoxic T cells (as identified by CD8 expression) and the total T helper cell population (as identified by CD4 expression) from total CD3-expressing T cells did not differ between AAT-treated and control mice (Table 1). Within the CD4-positive helper T-cell-subset, CD25 expressing Tregs did not differ among the experimental groups as well (Table 1). However, Th1 helper cells (as identified by CXCR3 expression) were lower in recipients treated with AAT (vehicle vs. AAT group (% of gated): median 33.9 vs. 21.0, p < 0.01; Fig. 7a). Similarly, treatment with AAT resulted in a reduction of the proportion of Th17 cells (as identified by CCR6 expression) from CD4-expressing helper T cells (vehicle vs. AAT group (% of gated): median 3.7 vs. 1.6, p < 0.01; Fig. 7b).

**Table 1:** Recipient blood leukocytes on postoperative day 3 (% of gated)

	Control	AAT	p value
Neutrophils	10.3 ± 5.3	7.0 ± 3.5	0.30
B cells	66.1 ± 17.6	72.3 ± 6.9	0.78
CTC	37.5 ± 4.2	34.5 ± 5.0	0.30
All Th cells	40.0 ± 5.0	42.8 ± 5.8	0.80
Tregs	17.9 ± 4.6	16.9 ± 3.5	0.61
Th1	34.4 ± 5.8	18.1 ± 4,7	0.001
Th17	3.8 ± 0.7	1.4 ± 1.2	0.04

*CTC* cytotoxic T cells, *Th* T helper, *Tregs* regulatory T cells

## Discussion

Experimental in vivo models which, at least in part, replicate human LuTx, can facilitate the translation of experimental findings into clinical settings. Therefore, to gain better knowledge about any putative benefits of AAT therapy in LuTx field, we performed orthotopic single lung transplantation in the BALB/CJ (donors) to C57BL/6 (recipients) mouse strain combination. To investigate the effect of AAT, we sacrificed the mice 3 days after LuTx, because the half-life of AAT is 4–5 days [11], and acute rejection in this experimental model is already visible on postoperative day 3 [30].

Both mouse strains are inbred strains, and their combination is a full major histocompatibility complex (MHC) mismatch resulting in acute rejection. This mouse model for acute allograft rejection is very useful not only because its well mimics human lung transplantation, but also because its allows to collect biological samples necessary to study both immunological and non-immunological aspects of lung transplant [31]. Nevertheless, only few laboratories have published data using this model, which is likely due to the technical complexity of the surgical technique and perioperative complications limiting recipient mice survival [32]. In our experience, the surviving rate after this orthotopic LuTx is only 60%, because of the pathophysiologic changes in pulmonary parenchyma after transplantation as well as due to the technical failures [31, 32]. Obviously, these facts explain why our findings are based on relatively small number of animals. On the other hand, despite the technical difficulties, orthotopic lung transplantation mouse model is highly reproducible and provides a great opportunity to explore new therapies.

Here we injected human plasma AAT preparations (5 mg) to the recipients prior to LuTx subcutaneously and immediately after the LuTx intraperitoneal. No other immune suppressive drugs were administered. Three days after the transplantation, when first robust histopathological changes typical for acute rejection are visible, the mice were sacrificed, and biological samples were assessed. Our results revealed that AAT, at a concentration used, can inhibit acute lung rejection after LuTx. Even though in AAT treated mice serum and lung levels of AAT remained higher 3 days after LuTx, there was no significant difference in the proportion of neutrophil granulocytes, B cells, and the total T helper cells between AAT treated and non-treated mice. Serum levels of CXCL1 (KC) did not differ between AAT treated and non-treated control mice as well. The chemokines stimulate the migration of leucocytes from the periphery into the allograft, for example IL-8 is an important activator and recruiter of neutrophils to areas of lung injury [33]. However, clinical studies show that the levels of IL-8 do not correlate with severity of acute lung rejection [34]. In agreement, results from our model show that AAT therapy does not affect neutrophil numbers as well as the levels of IL-8. Neutrophils have dynamic behavior, they can promote graft inflammation but also induce the expression of anti-inflammatory molecules in other myeloid cells [35], promote angiogenesis [36] and subsets of neutrophils can inhibit T cell activation [37]. It cannot be excluded, that early after LuTx neutrophils may protect the allograft from injury and promote tolerance. Future more detail studies will be required to determine the effects of AAT therapy on neutrophil subsets in LuTx recipients.

Although neutrophils may play a critical role early after LuTx, uncontrolled release of neutrophil elastase and other proteases may strongly contribute to tissue damage. Indeed, serum anti-elastase activity was significantly higher in AAT treated mice as compared to the non-treated controls. This finding was expected because circulating AAT is a major inhibitor of elastase [38]. According to previous studies, AAT also regulates human neutrophil degranulation [39]. Unfortunately, we are unable to tell if AAT affected neutrophil degranulation in our model. Firstly, the frequency of neutrophils in mice is lower compared to humans, mice neutrophils express different receptors and their granule content differ from that in humans [40]. For example, mice do not express human Fc receptor for IgA (FcαRI, CD89), which is involved in human neutrophil oxidative burst, cytokine release, and phagocytosis [41]. Therefore, neutrophil activation (degranulation) responses do not occur in the same way in humans and mice. Secondly, we primarily focused on neutrophil numbers and serum anti-elastase activity but not on specific neutrophil functions, and these remain to be addressed in future studies.

In organ transplantation, Th1 cells are associated with rejection whereas recent data have more specifically implicated IL-17 and Th17 cells in the development of rejection after LuTx in both animal models and in humans [6, 42, 43]. Data from our model show that the proportion of Th1 cells are significantly lower in lung recipients treated with AAT preparations. Specifically, treatment with AAT resulted in a significant reduction of the proportion of Th17 cells. Some authors suggest that in organ transplantation, including the lung, ischemia–reperfusion injury induces apoptosis, which might be the initial insult to induce differentiation of alloreactive Th17 cells [44]. In accordance, we found that the transplanted lungs of controls show a greater number of cleaved caspase-3 positive cells than AAT treated mice. Caspase-3 is a member of cysteine protease family and an important mediator of apoptosis [45]. According to previous studies, AAT can directly bind to caspase-3 [45] and inhibit its activity. Hence, reduced lung allograft rejection in AAT-treated recipients most likely linked to AAT property to inhibit caspase-3-mediated cell apoptosis, which indirectly reduces Th17 cell counts and a risk for acute rejection.

It is important to notify, that under baseline conditions serum AAT levels in mice are about 3–4 mg/ml [46]. Because AAT is an acute phase protein and its levels increase by 3–fourfold transiently during various inflammatory conditions [11], we investigated only early effects of AAT after LuTx, and we chose to treat lung recipients with constant doses (5 mg) of AAT therapy prior and after LuTx. We selected these doses of AAT based on the knowledge from AAT therapy in humans and other experimental models [47]. However, it cannot be excluded that higher doses of AAT might have produced even better effects, and this warrants further studies.

This study has numerous limitations. Among them is the small number of experimental animals investigated, which is mainly due to the technical difficulties inherent to lung transplantation in the mouse. Therefore, we only investigated one postoperative point in time, although it would be of interest to know if treatment with AAT also improves the long-term survival of pulmonary allografts. On the other hand, acute rejection is a major risk factor of chronic rejection after LuTx. We report a relatively high failure rate causing intraoperative death. The technique of transplantation consists of several critical steps, such as the anastomoses of blood vessels and the main bronchus using the cuff technique. These steps either fully succeed and result in properly perfused and ventilated grafts or result in intraoperative death of the recipient. Another point of criticism may be the use of human AAT to treat mice. However, it has been shown before that human AAT is active in mice [48]. The big advantage of using human AAT over murine AAT produced for research purposes is its availability in standardized high-quality preparations, which are endotoxin free and approved for clinical application. Further studies about the effect of AAT against chronic rejection are expected.

## Conclusions

Taken together, calcineurin inhibitors are used as main immunosuppressors after LuTx. These medicaments suppress T cells activity and prevent acute rejection but also can cause various complications [9, 10]. Results from our model show that AAT therapy brings a different mechanism against acute LuTx rejection. It seems that AAT inhibits apoptosis and affects Th cell subsets favoring LuTx recipient. Here we also would like to point out, that lung transplantation in AAT deficient patients does not resolve the inherited deficiency of AAT that originally led to the development of COPD/emphysema. Therefore, following transplantation, patients remain at risk of redeveloping emphysema and related clinical consequences, and some scientists have proposed that AAT therapy after transplantation may be beneficial [15]. We previously reported that AAT deficient patients, who received AAT therapy prior to but not after LuTx, show worse survival rates relative to AAT deficiency without prior augmentation or to AAT sufficient COPD patients [49]. It is possible that AATD patients, who receive AAT therapy prior to LuTx, have more profound illness, and therefore therapy continuation for a short period following transplantation could positively impact the post-transplant course. In support of this assumption, our current experimental data show, that AAT therapy given directly after LuTx can be beneficial as anti-inflammatory and immunomodulatory. We aim to continue studies on the effects and molecular mechanisms of AAT not only during acute but also during chronic LuTx rejection, i.e., BOS.

## Data Availability

All data generated or analysed during this study are included in this published article.

## Abbreviations

AAT: Alpha1-antitrypsin
BOS: Bronchiolitis obliterans syndrome
CLAD: Chronic lung allograft dysfunction
DAB: 3,3′-Diaminodbenzidine
EVOP: Ex vivo organ perfusion
HE: Hematoxylin–eosin
HRP: Horseradish peroxidase
ISHLT: International Society of Heart and Lung Transplantation
IRI: Ischemia–reperfusion-induced lung injury
LuTx: Lung transplantation
MHC: Major histocompatibility complex
OLTx: Orthotopic single lung transplantation
PBS: Phosphate buffered salt solution
PGD: Primary graft dysfunction
Th: T helper
T regs: Regulatory T cells
